# The re-emergence of influenza following the COVID-19 pandemic in Victoria, Australia, 2021 to 2022

**DOI:** 10.2807/1560-7917.ES.2023.28.37.2300118

**Published:** 2023-09-14

**Authors:** Catherine GA Pendrey, Janet Strachan, Heidi Peck, Ammar Aziz, Jean Moselen, Rob Moss, Md Rezanur Rahaman, Ian G Barr, Kanta Subbarao, Sheena G Sullivan

**Affiliations:** 1 WHO Collaborating Centre for Reference and Research on Influenza, Royal Melbourne Hospital, at the Peter Doherty Institute for Infection and Immunity, Melbourne, Australia; 2 National Centre for Epidemiology and Population Health, Australian National University, Canberra, Australia; 3 Communicable Diseases, Health Protection Branch, Public Health Division, Department of Health, Victoria, Melbourne, Australia; 4 School of Population and Global Health, University of Melbourne, Melbourne, Australia; 5 Department of Immunology and Microbiology, University of Melbourne, at the Peter Doherty Institute for Infection and Immunity, Melbourne, Australia; 6 Department of Infectious Diseases, University of Melbourne, at the Peter Doherty Institute for Infection and Immunity, Melbourne, Australia

**Keywords:** Influenza, bottleneck, phylogenetics, first few hundred, antigenic drift, university, travel

## Abstract

**Background:**

COVID-19 pandemic mitigation measures, including travel restrictions, limited global circulation of influenza viruses. In Australia, travel bans for non-residents and quarantine requirements for returned travellers were eased in November 2021, providing pathways for influenza viruses to be re-introduced.

**Aim:**

We aimed to describe the epidemiological and virological characteristics of the re-emergence of influenza in Victoria, Australia to inform public health interventions.

**Methods:**

From 1 November 2021 to 30 April 2022, we conducted an epidemiological study analysing case notification data from the Victorian Department of Health to describe case demographics, interviewed the first 200 cases to establish probable routes of virus reintroduction and examined phylogenetic and antigenic data to understand virus diversity and susceptibility to current vaccines.

**Results:**

Overall, 1,598 notifications and 1,064 positive specimens were analysed. The majority of cases (61.4%) occurred in the 15–34 years age group. Interviews revealed a higher incidence of international travel exposure during the first month of case detections, and high levels of transmission in university residential colleges were associated with return to campus. Influenza A(H3N2) was the predominant subtype, with a single lineage predominating despite multiple importations.

**Conclusion:**

Enhanced testing for respiratory viruses during the COVID-19 pandemic provided a more complete picture of influenza virus transmission compared with previous seasons. Returned international travellers were important drivers of influenza reemergence, as were young adults, a group whose role has previously been under-recognised in the establishment of seasonal influenza epidemics. Targeting interventions, including vaccination, to these groups could reduce future influenza transmission.

**Key public health message**

**What did you want to address in this study?**

Influenza was effectively absent from circulation in Australia from March 2020 to October 2021 as a result of international travel restrictions and quarantine requirements implemented in response to the COVID-19 pandemic. As these restrictions eased and travel recommenced in November 2021, we studied the re-occurrence of influenza in Victoria, Australia, to better understand how influenza spreads within populations to inform future public health responses.

**What have we learnt from this study?**

International travellers played an important role in introducing influenza viruses that then spread progressively in the community, leading to the establishment of local outbreaks and a seasonal epidemic. Once viruses were introduced, how much they spread in the community was highly variable. Adolescents and young adults made up a high proportion (54.4%) of early cases and the spread of influenza was possibly accelerated in universities.

**What are the implications of your findings for public health?**

Enhanced testing during the pandemic provided a more complete picture of influenza spread compared with prior seasons. In particular, this study highlighted the previously under-recognised role of young adults in promoting the spread of influenza. Targeting vaccination and other public health interventions to international travellers and young adults has the potential to reduce the spread and burden of influenza in Australia and comparable countries.

## Introduction

Although recently overshadowed by the impact of the COVID-19 pandemic, influenza remains an important cause of global morbidity and mortality. Seasonal influenza is estimated to cause 290,000–650,000 respiratory deaths each year, not accounting for cardiovascular and other non-respiratory deaths attributable to influenza [1].

In temperate climates, influenza epidemiology is characterised by seasonal epidemics during the cooler months, while in tropical regions outbreaks can occur year-round [2]. However, measures taken to control the COVID-19 pandemic during 2020 and 2021 resulted in unprecedented global suppression of circulating influenza viruses [3,4]. In the World Health Organization (WHO) European Region there was a 99.8% reduction in the number of specimens testing positive for influenza from October 2020 to February 2021 despite high levels of influenza testing. This reduction followed the introduction of strict COVID-19 pandemic control measures (e.g. mask wearing and limitations on gatherings) [5]. Similar patterns were seen in countries around the world [3] as influenza outbreaks were largely confined to parts of Asia and West Africa during this period [6].

In Australia, as part of the COVID-19 pandemic response the federal government introduced progressive travel restrictions beginning late February 2020 and culminating in border closure to all non-residents on 20 March 2020. In addition, a 14-day mandatory quarantine for returning Australian residents in supervised facilities (principally hotels) was introduced on 28 March 2020 [7]. Initiation of travel restrictions coincided with a dramatic reduction in influenza in Australia [8]. Only 33 of 60,031 specimens tested in Australia were positive for influenza between April and July 2020 [3], and between April 2020 and October 2021, confirmed cases were largely confined to quarantined travellers [8].

The reduction in global influenza transmission was accompanied by a decrease in genetic diversity, also referred to as bottlenecks, and marked geographic differentiation. Bottlenecks occurred for A(H1N1), A(H3N2) and B/Victoria viruses [4], and in the most extreme example, B/Yamagata was not detected after April 2020 [9]. Influenza viruses isolated from regions separated by travel restrictions in West Africa demonstrated genetic differentiation that mirrored the geographic boundaries of travel restrictions in this region [4].

As COVID-19 restrictions eased around the world, influenza activity began to increase in both the northern and southern hemispheres [10]. On 1 November 2021, Australia lifted quarantine requirements for COVID-19-vaccinated travellers entering into some jurisdictions, including Victoria [11]. The reopening of national borders and reinvigoration of international travel provided an opportunity for influenza viruses to recirculate [12]. These unique circumstances provided an ideal setting to study the establishment of seasonal influenza epidemics. We aimed to prospectively characterise the epidemiological and virological dynamics of the reemergence of influenza in Victoria to enhance understanding of seasonal epidemics in Australia and comparable countries and inform future public health responses.

## Methods

### Data source and case notifications

We describe the reintroduction of influenza to Victoria for the 6-month period between 1 November 2021, when quarantine-free international travel resumed, and 30 April 2021, by which time localised transmission in Victoria was evident. Victoria is located in the southern temperate region of Australia and has a population of 6.5 million which predominantly resides in Melbourne, a city of 4.9 million [13]. Influenza is notifiable in Victoria (and throughout Australia) with all cases of laboratory-confirmed influenza required to be notified to the Victorian Department of Health (DoH) by the diagnosing laboratories under the Public Health and Wellbeing Regulations 2019 [14]. Under an enhanced surveillance protocol that was in place from 1 November 2021 until 30 April 2022, laboratories were requested to send influenza-positive specimens to the World Health Organization Collaborating Centre for Reference and Research on influenza (WHO CCRRI) in Melbourne for further virus characterisation, and the first 200 confirmed cases were contacted for case interviews, as described below.

Case notification data for laboratory-confirmed influenza were extracted from the DoH Public Health Event Surveillance System. These included basic demographic information (age, sex and residential location) and influenza sample information (collection date, type and subtype). The unit of residential location used was Statistical Area Level 1 (SA1). These are unique geographic areas with populations of 200–800 people used by the Australian Bureau of Statistics [15]. Residential SA1 was used to approximate the socioeconomic position of cases using 2016 Australian Census data and the index of relative social advantage and disadvantage (IRSAD) [16]. Residential SA1 was also used to determine case remoteness, which was classified based on distance from populated centres using five categories (major cities, inner regional, outer regional, remote and very remote) according to the Australian Statistical Geography Standard [15]. Data were examined for demographic trends (age, sex, socioeconomic position and remoteness).

To contextualise influenza case detections within the ongoing COVID-19 pandemic, case notifications were compared with COVID-19 case notification numbers using data accessed from publicly available data on the Victorian DoH website [17]. Descriptive analyses were performed using R software version 4.1.2 (R Foundation, Vienna, Austria).

### Antigenic and genetic analyses

Specimens testing positive for influenza virus were forwarded to the WHO CCRRI for genetic and antigenic characterisation. Virus culture was attempted for all specimens in MDCK-SIAT1 cells (Madin-Darby canine kidney cells transfected with human 2,6-sialyltransferase) [18]. Antigenic similarity to the 2022 southern hemisphere seasonal influenza vaccine viruses was assessed by haemagglutination inhibition (HI) assay as previously described [19]. This assay measured the ability of post infection ferret antisera raised against representative viruses from the 2022 southern hemisphere vaccines - egg-grown A/Victoria/2570/2019 (H1N1)pdm09-like virus and cell-grown A/Darwin/6/2021 (H3N2)-like virus - to inhibit viral binding of red blood cells [9].

Sequencing was performed on the haemagglutinin (HA) gene from virus isolates, or original specimens if an isolate was not available, using the Sanger chain termination method of DNA sequencing [20] or Illumina iSeq (Illumina, Melbourne, Australia) as previously described [21]. Phylogenetic analysis and tree construction was undertaken using the maximum likelihood method and Augur pipeline [22], which uses IQTree [23] for constructing and bootstrapping (-B 1000 -alrt 1000) the phylogenetic tree (GTR model), then visualised using the R package ggtree [24].

### Case interviews

We identified the first 200 confirmed cases of influenza notified to the DoH after 1 November 2021 for interview. This approach was based on the First Few X protocol, originally used in the 2009 pandemic [25] and later adopted by the WHO [26] to characterise the epidemiological, clinical and virological properties of emerging respiratory pathogens through follow up of early cases and contacts. From 31 March to 10 May 2022, we attempted to contact each confirmed case for interview. All interviews were conducted by investigator CP via telephone. The standard DoH influenza interview form was used, which includes questions about vaccination status, risk factors for severe disease (including medical co-morbidity and pregnancy) and exposure to travel, agricultural settings and influenza cases. In addition, we included questions on pathways to access influenza testing. Following participant feedback during the first 30 interviews, a question was added on whether individuals had received their influenza test results. Clusters were identified based on epidemiological linkages (common exposure sites) and compared with phylogenetic data, where available, to identify potential transmission events.

## Results

**Figure 1:**
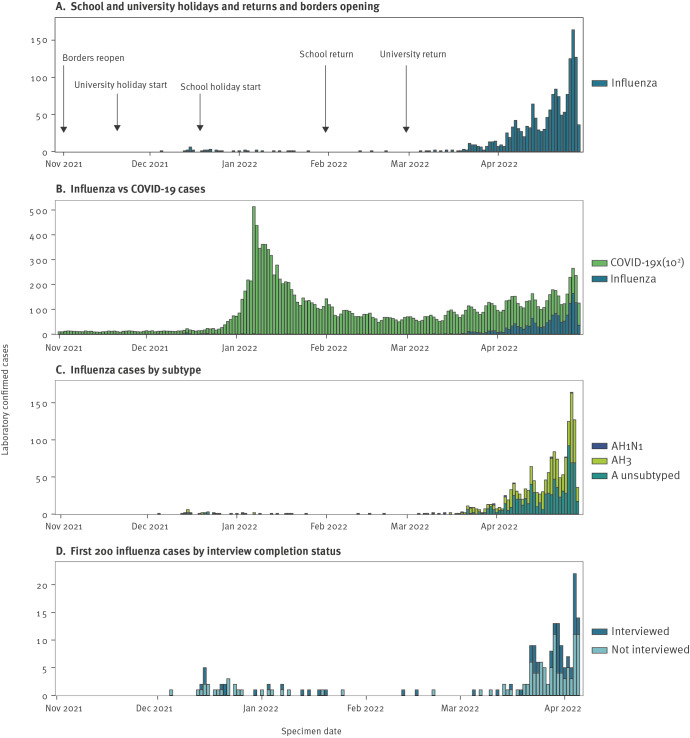
Laboratory-confirmed influenza cases showing (A) school and university holidays and returns and borders reopening; (B) influenza cases compared with COVID-19 cases; (C) influenza cases by subtype; (D) first 200 influenza cases by interview completion status, Victoria, Australia, 1 November 2021–30 April 2022

The first influenza notification was on 5 December 2021. Numbers remained low over the Australian summer (December 2021–February 2022), with 41 notifications, including only three in February 2022 (Figure 1A). This low level of influenza activity coincided with the COVID-19 epidemic that occurred from mid-December 2021 to February 2022 (Figure 1B). Regular influenza notifications progressively increased during March 2022, with a sustained upsurge that continued into April 2022. By the end of April, 1,598 laboratory-confirmed cases had been notified to DoH, all diagnosed as influenza A. Based on information submitted by diagnostic laboratories, subtype was only available for 87 cases (5.4%), of which 85 (97.7%) were A(H3N2) and two (2.3%) were A(H1N1)pdm09. By comparison, there were 1,478,163 COVID-19 cases in Victoria over the study period (1 November 2021 to 30 April 2022) (Figure 1B).

Panel A. Victorian summer school holidays occurred from 17 December 2021 to 31 January 2022 and university summer holidays occurred from mid-late November 2021 to 27 February 2022, with some variation between programs and institutions. Source: Victorian Department of Health.

Panel B. Source: Victorian Department of Health.

Panel C. Source: Victorian Department of Health and WHO Collaborating Centre for Reference and Research on Influenza.

Panel D: Daily influenza case notifications for first 200 case notifications 1 November 2021 to 6 April 2022. Source: Victoria Department of Health and study interview data.

**Figure 2:**
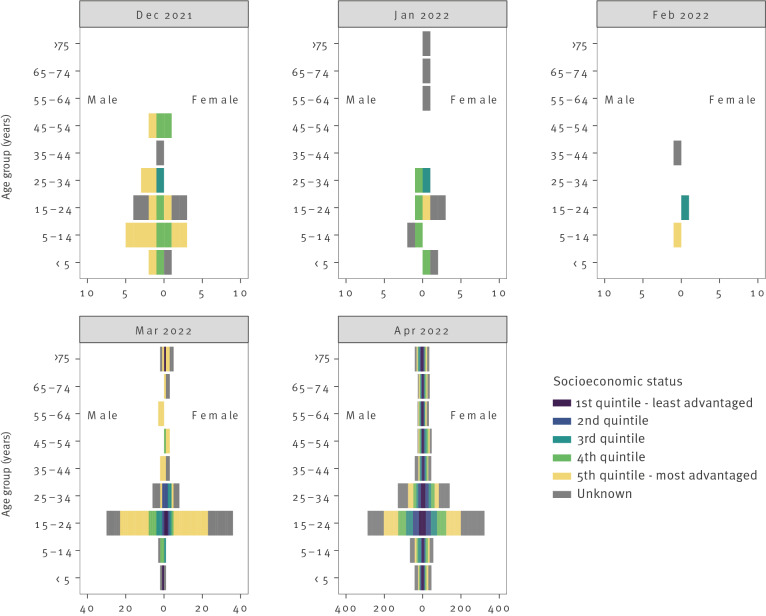
Demographic characteristics of influenza cases by month of infection. Victoria, Australia, 1 November 2021–30 April 2022 (n = 1,598)

There was a slight predominance of female cases (Figure 2). Nearly half of the 1,598 cases were young adults aged 15–24 years (22.6% aged 20–24 years and 20.7% aged 15–19 years, Supplementary Table S1). Individuals in age groups at increased risk of severe influenza comprised 15.4% of cases: 6.0% < 5 years, and 9.4% ≥ 65 years. In March 2022, when cases began to increase, the 20–24-year-old age group predominated with 41/108 cases (38.0%), followed by 15–19-year-olds with 25/108 cases (23.1%). In April 2022, the predominance of those aged 15–24 years partially abated as the relative proportion of cases increased in all other age groups, except individuals aged ≥ 75 years.

Socioeconomic status was determined according to the Index of Relative Social Advantage and Disadvantage (IRSAD), based on location of residence. Source: Victorian Department of Health and Australian Bureau of Statistics [16].

Of the 1,087 cases with available remoteness area classification, 917 (84.4%) occurred in major cities, 144 (13.2%) in inner regional areas and 26 (2.4%) in outer regional areas. Of the 1,058 cases with an available socioeconomic indicator, 140 (13.2%) were in the lowest quintile for Victoria and 354 (33.5%) were in the highest. The skew towards relative advantage was more pronounced before the increase in cases in April 2022. From November 2021 to March 2022, 61/98 (62.2%) cases lived in the highest quintile areas while only 4/98 (4.1%) lived in the lowest quintile areas.

### Antigenic analysis

From 1 November 2021 to 30 April 2022, 1,064 positive influenza samples were sent to the WHO CCRRI from Victorian diagnostic laboratories. The age and sex distribution of individuals with positive samples was similar to DoH notification data as displayed in Supplementary Table S2. All samples were influenza A, with no influenza B viruses identified (Figure 1C). Subtype was available for 714/1,064 samples (67.1%), of which 699 (97.9%) were A(H3N2) and just 15 (2.1%) were A(H1N1)pdm09.

Of 1,064 samples, HI was performed on 645 (60.6%) virus isolates. Of the 633/645 (98.1%) that were A(H3N2) isolates, 607 (95.9%) were antigenically similar to A/Darwin/6/2021. All 12 A(H1N1)pdm09 virus isolates demonstrated a similar antigenic profile to the A/Victoria/2570/2019 virus.

### Genomic sequencing

Of all specimens sent to the WHO CCRRI, whole genome sequencing results were available for 192 (18.0%). Of 180 A(H3N2) viruses, 179 (99.4%) were of the same HA genetic clade (3C.2a1b.2a.2) as the vaccine virus A/Darwin/6/2021, with the remaining viruses belonging to the 3C.2a1b.1a clade [9]. All 12 A(H1N1) influenza viruses sequenced were of the same HA clade (6B.1A.5a.2) as the vaccine virus A/Victoria/2570/2019 data are shown in Supplementary Table S3.

**Figure 3:**
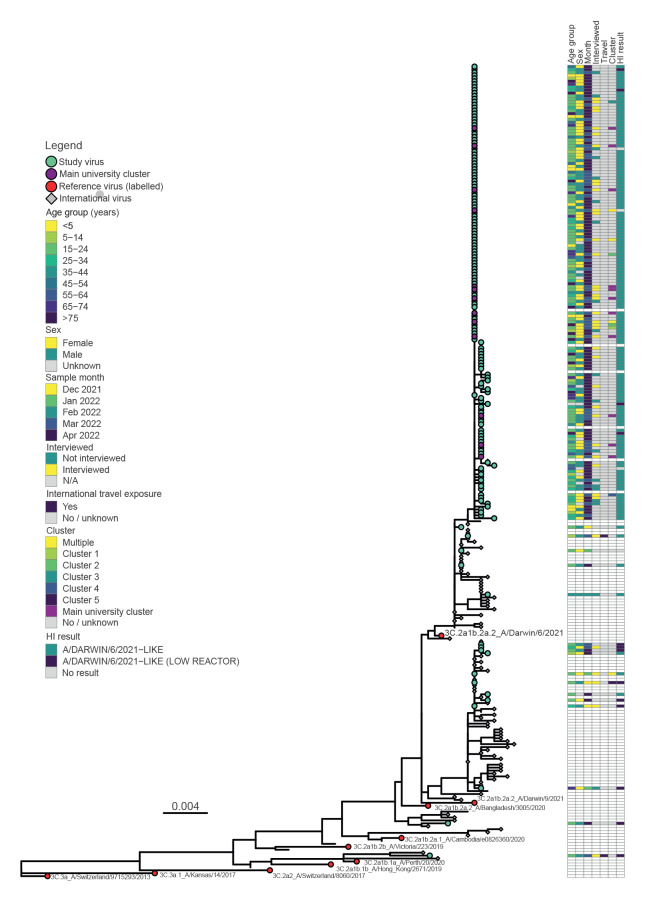
Phylogenetic trees of A(H3N2) influenza viruses, HA gene. Victoria, Australia, 1 November 2021–30 April 2022

A HA phylogenetic tree was constructed using 175/192 A(H3N2) viruses, with 17 excluded due to incomplete sequences and low depth coverage. The phylogenetic tree revealed a predominant genetic group within the HA 3C.2a1b.2a.2 clade with high levels of homogeneity, indicating a probable point source outbreak (Figure 3).

HI: haemagglutination inhibition assay.

For Global Initiative on Sharing All Influenza Data (GISAID) accession numbers and information available for viruses sequenced in this study, see Supplementary Table S3. For GISAID accession numbers for international and reference influenza viruses, see Supplementary Table S4.

### Case interviews

The first 200 case notifications were reported to DoH between 5 December 2021 and 6 April 2022. Of these, 178 (89.0%) had available contact information and 124 (62.0%) were successfully contacted for interview (Figure 1D). There were no significant differences in age and sex distribution between those who were and were not interviewed (Supplementary Table S5).

Of those who were interviewed, the median age was 20.9 years (interquartile range 18.8–27.6) (Table). There were approximately even numbers of male and female cases with the vast majority (116/124, 93.5%) living in major cities, and the remainder in inner regional areas.

**Table:** Characteristics of interviewed influenza cases, Victoria, Australia, 1 Nov 2021–6 Apr 2022

Interview cases	Dec n = 17		Jan n = 7		Feb n = 1		Mar n = 62		April n = 37		Total n = 124	
	n	%	n	%	n	%	n	%	n	%	n	%
Proportion of cases interviewed	17/25	68.0	7/13	53.8	1/3	33.3	62/95	65.3	37/64	57.8	124/200	62.0
Age (median years)	18.9		16.3		9.2		21.3		22.9		20.9	
Interquartile range	13.4–21.2		11.6–23.8		9.2–9.2		19.1–29.4		19.2–28.5		18.8–27.6	
Age < 5 years	2	11.8	1	14.3	0	0	2	3.2	1	2.7	6	4.8
Age ≥ 65 years	0	0	0	0	0	0	7	11.3	0	0	7	5.6
Sex												
Male	12	70.6	3	42.9	1	100	26	41.9	16	43.2	58	46.8
Female	5	29.4	4	57.1	0	0	36	58.1	21	56.8	66	53.2
Rurality												
Major city	17	100	7	100	1	100	60	96.8	31	83.8	116	93.5
Inner regional	0	0	0	0	0	0	2	3.2	6	16.2	8	6.5
Influenza vaccination 2021–2022^a^	5	29.4	1	14.3	0	0	21	33.9	15	23.4	43	34.7
Chronic disease any	3	17.6	3	42.9	0	0	20	32.3	15	23.4	41	33.1
Respiratory	2	11.8	2	28.6	0	0	12	19.4	8	12.5	24	19.4
CVD	1	5.9	0	0	0	0	5	8.1	1	1.6	4	3.2
Immunosuppression	0	0	0	0	0	0	4	6.5	1	1.6	5	4.0
Other	1	5.9	1	14.3	0	0	9	14.5	6	9.4	15	12.1
Pregnancy	0	0	0	0	0	0	0	0	1	2.7	1	0.8
International exposure ≤ 1 week before onset												
International travel	6	35.3	0	0	0	0	2	3.2	0	0	8	6.5
Contact with international traveller	0	0	1	14.3	0	0	4	6.5	0	0	5	4.0
Interstate^b^ exposure ≤ 1 week before onset												
Interstate travel	0	0	0	0	0	0	8	12.9	0	0	8	6.5
Contact with interstate traveller	0	0	0	0	0	0	2	3.2	3	4.7	5	4.0
Agricultural exposure	0	0	0	0	0	0	1	1.6	2	3.1	3	2.4

CVD: cardiovascular disease.

^a^ All vaccinations administered in 2021 or ≤ 1 week before onset of influenza infection in 2022.

^b^ Interstate refers to travel from a state or territory of Australia, other than Victoria.

Cases were interviewed about exposure within one week of symptom onset. Of the 124 interviewed cases, eight (6.5%) reported recent international travel, including six in December 2021. An additional five cases reported close contact with a returned international traveller, one in January 2022 and four in March. Individuals reported international travel exposure from Europe, Horn of Africa, the Middle East, North America, Pacific Islands, South Asia and South East Asia. December 2021 was the month with the highest proportion of interviewed cases reporting a history of exposure to international travel (6/17 interviewed cases). We calculated credible intervals across a range of probabilities (50–90%) for international travel exposure among all notifications based on the assumption that the proportion of interviewed cases who reported an international exposure is a binomial process (Supplementary Figure S1). Uncertainty of these estimates was higher in months with lower numbers of interviewed cases, most notably February. Thirteen cases (10.5%), all testing positive between 21 March and 6 April 2022, reported a history of interstate travel outside of Victoria or close contact with a returned interstate traveller. Only three cases reported spending time in an agricultural setting, including one reporting poultry contact.

Among interviewees, 41/124 (33.1%) reported having received an influenza vaccination in 2021 and 7/124 (5.6%) in 2022 (including one individual who was also vaccinated in 2021). The proportion vaccinated was 26/50 among those with risk factors for severe influenza illness and 20/74 among those without risk factors. The date of all 2022 vaccinations was reported as either unknown or ≤ 1 week before influenza onset. One third of interviewed cases reported having a chronic medical condition. Respiratory conditions were the most common (19.4%) and included a diagnosis of asthma in all cases.

Regarding health service pathways to accessing influenza testing, primary care clinics were the most common location of first attendances (55/124, 45.1%) and presentations (66/124, 54.1%), followed by emergency departments (first attendances: 30/124, 24.6%; presentations: 56/124, 45.9%) and COVID-19 testing clinics (first attendances: 23/124, 20.5%; presentations: 26/124, 21.3%) (Supplementary Table S6). Accordingly, more than 20% (26/124) of interviewed cases secondarily presented to an emergency department after attending another health service within their episode of illness to seek medical review. In total, 23/124 (18.5%) interviewed cases reported that they were not aware of their positive test result for influenza.

Through the limited contact and exposure history obtained, 56/124 cases (45.2%) were linked to nine clusters (Supplementary Figure S2). Remaining cases had no identified epidemiological links to other cases. There was one predominant cluster of 30 people associated with a single university campus that coincided with the increase in case detections in March 2022. Within this cluster, 12 cases lived in onsite university residential accommodation. During interviews, these cases described widespread transmission of influenza and acute respiratory illness other than COVID-19 on campus and especially within residential colleges. An additional seven cases were associated with other universities. Secondary transmission was reported within households and through social contacts. Other clusters with multiple cases were associated with music festivals, schools, private functions and hospitality venues.

Comparison of epidemiological and phylogenetic data indicated that all 13 cases with sequences identified as being within the main university epidemiological cluster were part of the predominant lineage within the A(H3N2) HA 3C.2a1b.2a2 clade that demonstrated high levels of genetic homogeneity (Figure 3). The two cases that had both virus sequence data and a history of interstate travel were also within this predominant genetic group. In contrast, neither of the two individuals with identified international travel exposure and sequences were within this genetic group. The A(H3N2) virus from one international traveller from the Horn of Africa was the only sequence identified within the HA 3C.2a1b.1a clade.

## Discussion

After an 18-month period of effective local elimination of influenza in Australia, mainly through travel restrictions and enforced quarantining in response to the COVID-19 pandemic, positive influenza case detections began shortly after the recommencement of quarantine-free international travel in November 2021. Case numbers remained low during the summer holiday period, coinciding with the SARS-CoV-2 Omicron variant (Phylogenetic Assignment of Named Global Outbreak (Pango) lineage designation BA.1) epidemic, and then showed a steady increase of A(H3N2) from late March 2022, which was possibly accelerated in universities. Increased access to multi-pathogen testing during the COVID-19 pandemic has enabled testing among groups not normally captured in surveillance data [27]. This has probably provided a more complete picture of influenza spread in the community in 2022 compared with previous years.

The 2022 influenza season in Australia progressed after our study ended and was characterised by an unusually early start, a sharp rise in cases followed by a rapid decline and a moderately severe epidemic season overall [28]. The season commenced before the annual influenza vaccination campaign in early April, by which time population immunity from the previous year’s vaccine was waning [29]. Influenza case numbers peaked in late May and early June 2022 during the Omicron BA.2 epidemic and proceeded to rapidly decline from late June. Expansion in influenza case numbers was accompanied by an increase in the diversity of influenza viruses in Victoria, including the first detections of B/Victoria viruses for the season and increased diversity in A(H3N2) viruses [9]. There was some variability across Australia. Several jurisdictions (New South Wales (NSW), Northern Territory and Western Australia) had higher proportions of A(H1N1) early in the season [12]. However, A(H3N2) was ultimately the predominant subtype in all jurisdictions, many in the same predominant genetic subgroup as observed in our study.

Consistent with previous studies, our findings highlighted the important role of travel-related importations in establishing seasonal influenza epidemics [30-32]. The re-opening of international borders preceded the Australian summer holidays, which occurred in Victoria from 17 December 2021 to 31 January 2022 for primary and secondary schools and mid-late November 2021 to 27 February 2022 for universities, with some variation between programs and institutions. The frequency of international arrivals among interviewed cases (6.5%) exceeded the rate observed in the Australian population over the same period, equivalent to 1.1% per month from December 2021–April 2022 [13,33]. We also identified a higher rate of international travel exposure earlier in the study period, 6/17 (35.3%) in December compared with 7/107 (6.5%) in January–April. Noting the limitations of surveillance [8], all except one case (who had returned from the Pacific Islands) were exposed to regions where there was detectable influenza activity at the time. Although numbers were small, together with the recommencement of cases after border opening, this suggests importations led to sustained local transmission. In contrast, cases with a history of interstate travel exposure were not detected until 4 months after borders re-opened, coinciding with the sustained increase in local case detections in March 2022. An Australian study investigating influenza cases in NSW during the 2018–19 summer also noted the importance of travel during summer epidemics. The authors showed that influenza cases were seven times more likely to report recent international travel exposure during the first 2 months of the study, compared with later in the summer [30]. Belderok et al. similarly identified the importance of travel-related importations in a European setting in a 2006–07 study, with 7.2% of short-term travellers to tropical and subtropical regions contracting influenza. In addition, half of those with symptomatic influenza infection were considered contagious on returning to the Netherlands [31].

Previous phylogenetic studies have determined that influenza virus circulation in Australia is seeded by multiple importations from the global virus population, followed by rapid local dissemination [32]. In our study, we observed multiple incursions, but overwhelming predominance of a single A(H3N2) subgroup within the 3C.2a1b.2a.2 clade. The genetically homogenous grouping could represent a point-source outbreak or multiple introductions of genetically similar viruses, as has previously been observed in university-based influenza surveillance [34]. Reduced transmission during the summer, competition with the first Omicron BA.1 epidemic and ongoing adherence with non-pharmaceutical interventions (NPIs) as well as incomplete sampling and stochastic transmission variability may all account for the limited observed onward transmission of some viruses [35].

Our study highlighted the potential importance of young adults in establishing seasonal influenza epidemics. During March 2022, as case numbers started to increase and universities returned to onsite teaching, almost 40% of cases occurred in adults aged 20–24 years. The influenza literature has traditionally emphasised the role of school-aged children in transmission, rather than young adults [36]. However, notable influenza outbreaks on university campuses since the relaxation of pandemic travel restrictions [37,38] and evidence from the COVID-19 pandemic that community-wide transmission was predicted by increased disease incidence among people aged < 25 years [39] underscore the potentially important—and previously underappreciated—role of young adults in transmission. Young adults have high levels of mobility and social interaction [40,41], but comparatively lower rates of influenza vaccination [42] and lower use of NPIs [43]. Younger individuals with less cumulative exposure to influenza may also have been disproportionately vulnerable to influenza infection after 2 years of no transmission [44].

Universities were identified in interviews as an important setting for transmission. Numerous university outbreaks also occurred in the United States (US) early in the post-pandemic reemergence of influenza, including a rapidly proliferating A(H3N2) outbreak at the University of Michigan [37,38]. Similarly in the pre-pandemic period, a longitudinal air sampling study from October 2016 to June 2018 found that increased detections of influenza viruses at a Hong Kong university campus preceded the seasonal epidemics that occurred during the study [45]. One modelling study concluded that university campuses are susceptible to rapid transmission and high attack rates during influenza outbreaks. University residential colleges increase risks as high-density accommodation and shared facilities promote inter-personal contact and constrain the capacity to limit transmission [41]. The return to university after the recommencement of international travel during the holiday period likely compounded the risk of outbreaks in Melbourne. From November 2021, there was a dramatic increase in the total number of overseas arrivals to Australia. Over the same period, the number of international students arriving in Australia to study at universities also rebounded [33].

The high proportion of young adults and international travellers in our study raises the prospect of targeting interventions to these groups to reduce the overall burden of influenza. Vaccination is the most practical means of mitigating influenza epidemics and is provided free in Australia under the National Immunisation Programme (NIP) to high-risk groups including children < 5 years old, adults ≥ 65 years old, individuals with specified chronic medical conditions, Aboriginal and Torres Strait Islander individuals and pregnant women [46]. Individuals who are not eligible may access influenza vaccination privately but at their own cost. In June 2022, in response to concern that rapidly rising influenza case numbers could overwhelm health systems already strained by the pandemic, eligibility for free influenza vaccination was expanded to all age groups in most Australian jurisdictions, including Victoria. In future seasons, maintaining expanded eligibility for subsidised vaccination and promoting vaccination among travellers could reduce the community burden of influenza. A similar approach, in which vaccination is provided free to school-age children, has been employed in the United Kingdom (UK) [47], Ireland [48], Western Australia [49] and Japan [50]. Marsh et al. also highlighted the importance of promoting vaccination among travellers, including revaccination for those vaccinated earlier in the season, to mitigate importations propagating outbreaks [30]. Specifically promoting vaccination and testing among young adults may also be an effective strategy to reduce overall community transmission. Likewise, maintaining some level of NPIs for acute respiratory illnesses has the potential to reduce influenza-related morbidity and mortality [51]. Social distancing 1.5 m, frequent hand washing, respiratory hygiene, wearing face masks, maintaining ventilation and staying home when unwell all continue to be recommended in Victoria [52]. However, compliance is low [53].

A key mitigation measure used in Australia during the COVID-19 pandemic was testing to guide isolation [54], with results communicated directly to those being tested rather than via clinicians as was previously the case. During our study it was apparent that some cases were not aware of their influenza diagnosis, which limits a case’s ability to isolate to protect others. Following this study, the government has worked with pathology providers to communicate influenza and other respiratory virus test results direct to consumers. As influenza is often viewed as more severe than the common cold, it is hoped that receiving positive results will increase the likelihood individuals adopt respiratory hygiene measures [43,55].

### Limitations

It is unlikely that our study captured all international importations of influenza, which would include asymptomatic and mild cases that were not tested for influenza. It is possible that under-ascertainment could have been greater early in the study period before broad recognition that influenza had returned. Genetic sequences were available for 192/1,064 (18.0%) of positive specimens. More complete sequencing may have helped to provide a richer understanding of transmission dynamics. Incomplete recollection of events limited our ability to identify epidemiological links between cases.

## Conclusions

We documented the reestablishment of endemic influenza in Victoria, Australia, after a prolonged absence of circulation. Our findings reinforce the importance of international travellers and highlight the role of young adults in promoting the spread of influenza viruses and the potential value of targeting interventions to these groups. As we have now reconnected globally, efforts to increase influenza vaccination uptake should continue to be prioritised, with increased focus on young adults and international travellers.

## Supplementary Data

748000 Supplementary Material
