# Supplementary material for: The re-emergence of influenza following the COVID-19 pandemic in Victoria, Australia, 2021 to 2022
Source: Euro Surveill. 2023 Sep 14;28(37):2300118. doi: 10.2807/1560-7917.ES.2023.28.37.2300118 (PMC10687983; doi:10.2807/1560-7917.ES.2023.28.37.2300118)
Supplement: Supplementary Material [file 23-00118_PENDREY_Supplement.pdf]

Supplementary material

This supplementary material is hosted by *Eurosurveillance* as supporting information alongside the article 'The re-emergence of influenza following the COVID-19 pandemic in Victoria, Australia, 2021 to 2022', on behalf of the authors, who remain responsible for the accuracy and appropriateness of the content. The same standards for ethics, copyright, attributions and permissions as for the article apply. Supplements are not edited by *Eurosurveillance* and the journal is not responsible for the maintenance of any links or email addresses provided therein.

Supplementary Table S1. Demographic characteristics of influenza cases, Victoria, Australia, 1 November 2021 – 30 April 2022.

	2021-12 (n=25)		2022-01 (n=13)		2022-02 (n=3)		2022-03 (n=108)		2022-04 (n=1449)		Total (n=1598)	
Sex	n	%	n	%	n	%	n	%	n	%	n	%
- Female	8	32.0	9	69.2	1	33.3	60	55.6	761	52.5	839	52.5
- Male	17	68.0	4	30.8	2	66.7	48	44.4	683	47.1	754	47.2
- Not stated	0	0.0	0	0.0	0	0.0	0	0.0	5	0.3	5	0.3
Age group (years)												
- <5	3	12.0	2	15.4	0	0.0	3	2.8	88	6.1	96	6.0
- 5-10	1	4.0	2	15.4	1	33.3	3	2.8	64	4.4	71	4.4
- 10-14	7	28.0	0	0.0	0	0.0	1	0.9	57	3.9	65	4.1
- 15-19	3	12.0	3	23.1	1	33.3	25	23.1	298	20.6	330	20.7
- 20-24	4	16.0	1	7.7	0	0.0	41	38.0	315	21.7	361	22.6
- 25-29	2	8.0	2	15.4	0	0.0	10	9.3	164	11.3	178	11.1
- 30-34	1	4.0	0	0.0	0	0.0	4	3.7	107	7.4	112	7.0
- 35-44	1	4.0	0	0.0	1	33.3	5	4.6	86	5.9	93	5.8
- 45-54	3	12.0	0	0.0	0	0.0	3	2.8	74	5.1	80	5.0
- 55-64	0	0.0	1	7.7	0	0.0	3	2.8	58	4.0	62	3.9
- 65-74	0	0.0	1	7.7	0	0.0	3	2.8	62	4.3	66	4.1
- ≥75	0	0.0	1	7.7	0	0.0	7	6.5	76	5.2	84	5.3
Rurality												
- Major City	17	68.0	6	46.2	2	66.7	77	71.3	815	56.2	917	57.4
- Inner Regional	2	8.0	0	0.0	0	0.0	2	1.9	140	9.7	144	9.0
- Outer Regional	0	0.0	0	0.0	0	0.0	0	0.0	26	1.8	26	1.6
- Unknown	6	24.0	7	53.8	1	33.3	29	26.9	468	32.3	511	32.0
Socioeconomic status [a]												
- 1st quintile	0	0.0	0	0.0	0	0.0	4	3.7	136	9.4	140	8.8
- 2nd quintile	0	0.0	0	0.0	0	0.0	5	4.6	154	10.7	159	10.0
- 3rd quintile	1	4.0	1	7.7	1	33.3	7	6.5	167	11.6	177	11.1
- 4th quintile	6	24.0	4	30.8	0	0.0	8	7.4	210	14.5	228	14.3
- 5th quintile	12	48.0	1	7.7	1	33.3	47	43.5	293	20.3	354	22.2
- Unknown	6	24.0	7	53.8	1	33.3	37	34.3	484	33.5	535	33.6

[a] Socioeconomic status according to the index of relative social advantage and disadvantage (IRSAD), based on location of residence, 1st quintile represents the least advantaged area and 5th quintile the most advantaged area.
Data source: Victorian Department of Health

Supplementary Table S2. Comparison between demographics of influenza cases reported to the Department of Health and cases with a specimen available for antigenic and genetic analysis at WHO CCRRI

	WHO CCRRI specimens (n=1064		Department of Health cases (n=1598		p-value[a]
Sex					0.66
- Female	419	39.4%	839	52.5%	
- Male	361	33.9%	754	47.2%	
- Unknown	284	26.7%	5	0.3%	
Age group					0.54
- <5	59	5.5%	96	6.0%	
- 5-14	82	7.7%	136	8.5%	
- 15-24	416	39.1%	691	43.2%	
- 25-34	188	17.7%	290	18.1%	
- 35-44	69	6.5%	93	5.8%	
- 45-54	51	4.8%	80	5.0%	
- 55-64	52	4.9%	62	3.9%	
- 65-74	54	5.1%	66	4.1%	
- ≥75	64	6.0%	84	5.3%	
- Unknown	29	2.7%	0	0.0%	

[a]p-values calculated with chi-squared tests, excluding unknown values. Based on inclusion of unknown values, p=<0.001 for both age and sex.

Supplementary Table S3. GISAID Accession numbers for influenza viruses sequenced, Victoria, Australia, 1 November 2021 – 30 April 2022

Isolate_Id	Isolate_Name	Clade	Subtype	Submitting_Lab	Authors	Originating_Lab
EPI_ISL_11499449	A/Victoria/2/2022	3C.2a1b.2a.2	A/H3N2	WHO CCRRI	This study	Monash Medical Centre
EPI_ISL_12004355	A/Victoria/24/2022	3C.2a1b.2a.2	A/H3N2	WHO CCRRI	This study	ST Vincent Hospital
EPI_ISL_12004532	A/Victoria/26/2022	3C.2a1b.2a.2	A/H3N2	WHO CCRRI	This study	Australian Clinical Laboratories
EPI_ISL_12004533	A/Victoria/27/2022	3C.2a1b.2a.2	A/H3N2	WHO CCRRI	This study	ST Vincent Hospital
EPI_ISL_12004534	A/Victoria/17/2021	3C.2a1b.2a.2	A/H3N2	WHO CCRRI	This study	Alfred Hospital
EPI_ISL_12206246	A/Victoria/6/2022	3C.2a1b.2a.2	A/H3N2	WHO CCRRI	This study	Alfred Hospital
EPI_ISL_12206250	A/Victoria/12/2022	3C.2a1b.2a.2	A/H3N2	WHO CCRRI	This study	Royal Melbourne Hospital
EPI_ISL_12206251	A/Victoria/10/2022	3C.2a1b.2a.2	A/H3N2	WHO CCRRI	This study	Alfred Hospital
EPI_ISL_12206253	A/Victoria/5/2022	3C.2a1b.2a.2	A/H3N2	WHO CCRRI	This study	Alfred Hospital
EPI_ISL_12206256	A/Victoria/16/2022	3C.2a1b.2a.2	A/H3N2	WHO CCRRI	This study	Royal Melbourne Hospital
EPI_ISL_12206257	A/Victoria/18/2022	3C.2a1b.2a.2	A/H3N2	WHO CCRRI	This study	Royal Melbourne Hospital
EPI_ISL_12206258	A/Victoria/17/2022	3C.2a1b.2a.2	A/H3N2	WHO CCRRI	This study	Royal Melbourne Hospital
EPI_ISL_12206259	A/Victoria/11/2022	3C.2a1b.2a.2	A/H3N2	WHO CCRRI	This study	Alfred Hospital
EPI_ISL_12206260	A/Victoria/15/2022	3C.2a1b.2a.2	A/H3N2	WHO CCRRI	This study	Monash Medical Centre
EPI_ISL_12206264	A/Victoria/4/2022	3C.2a1b.2a.2	A/H3N2	WHO CCRRI	This study	Alfred Hospital
EPI_ISL_12206274	A/Victoria/7/2022	6B.1A.5a.2	A/H1N1	WHO CCRRI	This study	WHO CCRRI
EPI_ISL_12206276	A/Victoria/13/2022	6B.1A.5a.2	A/H1N1	WHO CCRRI	This study	Monash Medical Centre
EPI_ISL_12206280	A/Victoria/9/2022	6B.1A.5a.2	A/H1N1	WHO CCRRI	This study	WHO CCRRI
EPI_ISL_12579823	A/Victoria/47/2022	3C.2a1b.2a.2	A/H3N2	WHO CCRRI	This study	St Vincent Hospital
EPI_ISL_12579824	A/Victoria/51/2022	3C.2a1b.2a.2	A/H3N2	WHO CCRRI	This study	Dorevtich Pathology
EPI_ISL_12579835	A/Victoria/52/2022	3C.2a1b.2a.2	A/H3N2	WHO CCRRI	This study	Dorevtich Pathology
EPI_ISL_12579850	A/Victoria/53/2022	3C.2a1b.2a.2	A/H3N2	WHO CCRRI	This study	Dorevtich Pathology
EPI_ISL_12579866	A/Victoria/54/2022	3C.2a1b.2a.2	A/H3N2	WHO CCRRI	This study	Dorevtich Pathology
EPI_ISL_12579868	A/Victoria/56/2022	3C.2a1b.2a.2	A/H3N2	WHO CCRRI	This study	ST Vincent Hospital
EPI_ISL_12579870	A/Victoria/66/2022	6B.1A.5a.2	A/H1N1	WHO CCRRI	This study	Royal Melbourne Hospital
EPI_ISL_12579871	A/Victoria/49/2022	3C.2a1b.2a.2	A/H3N2	WHO CCRRI	This study	Royal Melbourne Hospital
EPI_ISL_12579872	A/Victoria/50/2022	3C.2a1b.2a.2	A/H3N2	WHO CCRRI	This study	Dorevtich Pathology
EPI_ISL_12579875	A/Victoria/67a/2022	3C.2a1b.2a.2	A/H3N2	WHO CCRRI	This study	Royal Melbourne Hospital
EPI_ISL_12579876	A/Victoria/42/2022	3C.2a1b.2a.2	A/H3N2	WHO CCRRI	This study	Alfred Hospital
EPI_ISL_12579877	A/Victoria/62/2022	3C.2a1b.2a.2	A/H3N2	WHO CCRRI	This study	Monash Medical Centre
EPI_ISL_12579878	A/Victoria/123/2022	3C.2a1b.2a.2	A/H3N2	WHO CCRRI	This study	Royal Melbourne Hospital
EPI_ISL_12579879	A/Victoria/124/2022	3C.2a1b.2a.2	A/H3N2	WHO CCRRI	This study	Royal Melbourne Hospital
EPI_ISL_12579880	A/Victoria/67/2022	3C.2a1b.2a.2	A/H3N2	WHO CCRRI	This study	Royal Melbourne Hospital
EPI_ISL_12579881	A/Victoria/99/2022	3C.2a1b.2a.2	A/H3N2	WHO CCRRI	This study	Austin Health
EPI_ISL_12579882	A/Victoria/109/2022	3C.2a1b.2a.2	A/H3N2	WHO CCRRI	This study	Austin Health
EPI_ISL_12579883	A/Victoria/110/2022	3C.2a1b.2a.2	A/H3N2	WHO CCRRI	This study	Austin Health
EPI_ISL_12579887	A/Victoria/108/2022	6B.1A.5a.2	A/H1N1	WHO CCRRI	This study	ST Vincent Hospital
EPI_ISL_12579894	A/Victoria/33/2022	3C.2a1b.2a.2	A/H3N2	WHO CCRRI	This study	ST Vincent Hospital
EPI_ISL_12579895	A/Victoria/27/2022	3C.2a1b.2a.2	A/H3N2	WHO CCRRI	This study	Australian Clinical Laboratories
EPI_ISL_12579896	A/Victoria/68/2022	3C.2a1b.2a.2	A/H3N2	WHO CCRRI	This study	Royal Melbourne Hospital
EPI_ISL_12579897	A/Victoria/26/2022	3C.2a1b.2a.2	A/H3N2	WHO CCRRI	This study	Australian Clinical Laboratories
EPI_ISL_12579898	A/Victoria/32/2022	3C.2a1b.2a.2	A/H3N2	WHO CCRRI	This study	ST Vincent Hospital
EPI_ISL_12579899	A/Victoria/14/2022	3C.2a1b.2a.2	A/H3N2	WHO CCRRI	This study	Monash Medical Centre
EPI_ISL_12579902	A/Victoria/78/2022	3C.2a1b.2a.2	A/H3N2	WHO CCRRI	This study	Austin Health
EPI_ISL_12579903	A/Victoria/105/2022	3C.2a1b.2a.2	A/H3N2	WHO CCRRI	This study	ST Vincent Hospital
EPI_ISL_12579905	A/Victoria/107/2022	3C.2a1b.2a.2	A/H3N2	WHO CCRRI	This study	ST Vincent Hospital
EPI_ISL_12579908	A/Victoria/119/2022	3C.2a1b.2a.2	A/H3N2	WHO CCRRI	This study	ST Vincent Hospital
EPI_ISL_12579909	A/Victoria/85/2022	3C.2a1b.2a.2	A/H3N2	WHO CCRRI	This study	Monash Medical Centre
EPI_ISL_12579910	A/Victoria/120/2022	3C.2a1b.2a.2	A/H3N2	WHO CCRRI	This study	Austin Health
EPI_ISL_12579911	A/Victoria/121/2022	3C.2a1b.2a.2	A/H3N2	WHO CCRRI	This study	Austin Health
EPI_ISL_12579913	A/Victoria/130/2022	3C.2a1b.2a.2	A/H3N2	WHO CCRRI	This study	ST Vincent Hospital

EPI_ISL_12579914	A/Victoria/59/2022	3C.2a1b.2a.2	A/H3N2	WHO CCRRI	This study	Royal Chidrens Hospital
EPI_ISL_12579915	A/Victoria/44/2022	3C.2a1b.2a.2	A/H3N2	WHO CCRRI	This study	Monash Medical Centre
EPI_ISL_12579916	A/Victoria/64/2022	3C.2a1b.2a.2	A/H3N2	WHO CCRRI	This study	Monash Medical Centre
EPI_ISL_12579917	A/Victoria/63/2022	3C.2a1b.2a.2	A/H3N2	WHO CCRRI	This study	Monash Medical Centre
EPI_ISL_12579918	A/Victoria/73/2022	6B.1A.5a.2	A/H1N1	WHO CCRRI	This study	Alfred Hospital
EPI_ISL_12579919	A/Victoria/41/2022	3C.2a1b.2a.2	A/H3N2	WHO CCRRI	This study	Alfred Hospital
EPI_ISL_12579920	A/Victoria/43/2022	3C.2a1b.1a	A/H3N2	WHO CCRRI	This study	Monash Medical Centre
EPI_ISL_12579921	A/Victoria/125/2022	3C.2a1b.2a.2	A/H3N2	WHO CCRRI	This study	Royal Melbourne Hospital
EPI_ISL_12579922	A/Victoria/126/2022	3C.2a1b.2a.2	A/H3N2	WHO CCRRI	This study	Royal Melbourne Hospital
EPI_ISL_12579923	A/Victoria/79/2022	3C.2a1b.2a.2	A/H3N2	WHO CCRRI	This study	Royal Melbourne Hospital
EPI_ISL_12579924	A/Victoria/101/2022	3C.2a1b.2a.2	A/H3N2	WHO CCRRI	This study	WHO CCRRI
EPI_ISL_12579925	A/Victoria/76/2022	3C.2a1b.2a.2	A/H3N2	WHO CCRRI	This study	Monash Medical Centre
EPI_ISL_12579926	A/Victoria/22/2022	3C.2a1b.2a.2	A/H3N2	WHO CCRRI	This study	Royal Melbourne Hospital
EPI_ISL_12579931	A/Victoria/72/2022	3C.2a1b.2a.2	A/H3N2	WHO CCRRI	This study	Alfred Hospital
EPI_ISL_12579932	A/Victoria/71/2022	3C.2a1b.2a.2	A/H3N2	WHO CCRRI	This study	Alfred Hospital
EPI_ISL_12579933	A/Victoria/69/2022	3C.2a1b.2a.2	A/H3N2	WHO CCRRI	This study	Alfred Hospital
EPI_ISL_12579934	A/Victoria/74/2022	3C.2a1b.2a.2	A/H3N2	WHO CCRRI	This study	Alfred Hospital
EPI_ISL_12579935	A/Victoria/39/2022	3C.2a1b.2a.2	A/H3N2	WHO CCRRI	This study	Alfred Hospital
EPI_ISL_12579936	A/Victoria/20/2022	3C.2a1b.2a.2	A/H3N2	WHO CCRRI	This study	Royal Melbourne Hospital
EPI_ISL_12579937	A/Victoria/23/2022	3C.2a1b.2a.2	A/H3N2	WHO CCRRI	This study	Royal Melbourne Hospital
EPI_ISL_12579938	A/Victoria/58/2022	3C.2a1b.2a.2	A/H3N2	WHO CCRRI	This study	Monash Medical Centre
EPI_ISL_12579939	A/Victoria/57/2022	3C.2a1b.2a.2	A/H3N2	WHO CCRRI	This study	WHO CCRRI
EPI_ISL_12579942	A/Victoria/102/2022	3C.2a1b.2a.2	A/H3N2	WHO CCRRI	This study	WHO CCRRI
EPI_ISL_12579943	A/Victoria/77/2022	3C.2a1b.2a.2	A/H3N2	WHO CCRRI	This study	ST Vincent Hospital
EPI_ISL_12579944	A/Victoria/115/2022	3C.2a1b.2a.2	A/H3N2	WHO CCRRI	This study	Monash Medical Centre
EPI_ISL_12579945	A/Victoria/83/2022	6B.1A.5a.2	A/H1N1	WHO CCRRI	This study	Monash Medical Centre
EPI_ISL_12579946	A/Victoria/81/2022	3C.2a1b.2a.2	A/H3N2	WHO CCRRI	This study	Monash Medical Centre
EPI_ISL_12579947	A/Victoria/80/2022	3C.2a1b.2a.2	A/H3N2	WHO CCRRI	This study	Monash Medical Centre
EPI_ISL_12579948	A/Victoria/87/2022	3C.2a1b.2a.2	A/H3N2	WHO CCRRI	This study	Monash Medical Centre
EPI_ISL_12579949	A/Victoria/84/2022	3C.2a1b.2a.2	A/H3N2	WHO CCRRI	This study	Monash Medical Centre
EPI_ISL_12579950	A/Victoria/86/2022	3C.2a1b.2a.2	A/H3N2	WHO CCRRI	This study	Monash Medical Centre
EPI_ISL_12579951	A/Victoria/37/2022	3C.2a1b.2a.2	A/H3N2	WHO CCRRI	This study	Alfred Hospital
EPI_ISL_12579952	A/Victoria/113/2022	3C.2a1b.2a.2	A/H3N2	WHO CCRRI	This study	Monash Medical Centre
EPI_ISL_12579953	A/Victoria/111/2022	3C.2a1b.2a.2	A/H3N2	WHO CCRRI	This study	Monash Medical Centre
EPI_ISL_12579954	A/Victoria/112/2022	3C.2a1b.2a.2	A/H3N2	WHO CCRRI	This study	Monash Medical Centre
EPI_ISL_12579956	A/Victoria/70/2022	3C.2a1b.2a.2	A/H3N2	WHO CCRRI	This study	Alfred Hospital
EPI_ISL_12850597	A/Victoria/60/2022	6B.1A.5a.2	A/H1N1	WHO CCRRI	This study	WHO CCRRI
EPI_ISL_12850600	A/Victoria/412/2022	6B.1A.5a.2	A/H1N1	WHO CCRRI	This study	Royal Melbourne Hospital
EPI_ISL_12850879	A/Victoria/341/2022	3C.2a1b.2a.2	A/H3N2	WHO CCRRI	This study	ST Vincent Hospital
EPI_ISL_12850890	A/Victoria/1152/2022	3C.2a1b.2a.2	A/H3N2	WHO CCRRI	This study	WHO CCRRI
EPI_ISL_12850901	A/Victoria/371/2022	3C.2a1b.2a.2	A/H3N2	WHO CCRRI	This study	WHO CCRRI
EPI_ISL_12850911	A/Victoria/693/2022	3C.2a1b.2a.2	A/H3N2	WHO CCRRI	This study	Austin Health
EPI_ISL_12850922	A/Victoria/209/2022	3C.2a1b.2a.2	A/H3N2	WHO CCRRI	This study	Alfred Hospital
EPI_ISL_12850932	A/Victoria/232/2022	3C.2a1b.2a.2	A/H3N2	WHO CCRRI	This study	Alfred Hospital
EPI_ISL_12850942	A/Victoria/234/2022	3C.2a1b.2a.2	A/H3N2	WHO CCRRI	This study	Alfred Hospital
EPI_ISL_12850952	A/Victoria/273/2022	3C.2a1b.2a.2	A/H3N2	WHO CCRRI	This study	Alfred Hospital
EPI_ISL_12851004	A/Victoria/254/2022	3C.2a1b.2a.2	A/H3N2	WHO CCRRI	This study	Royal Melbourne Hospital
EPI_ISL_12851014	A/Victoria/410/2022	3C.2a1b.2a.2	A/H3N2	WHO CCRRI	This study	Royal Melbourne Hospital
EPI_ISL_12851024	A/Victoria/391/2022	3C.2a1b.2a.2	A/H3N2	WHO CCRRI	This study	Royal Melbourne Hospital
EPI_ISL_12851034	A/Victoria/437/2022	3C.2a1b.2a.2	A/H3N2	WHO CCRRI	This study	Royal Melbourne Hospital
EPI_ISL_12851045	A/Victoria/266/2022	3C.2a1b.2a.2	A/H3N2	WHO CCRRI	This study	Alfred Hospital
EPI_ISL_12851076	A/Victoria/278/2022	3C.2a1b.2a.2	A/H3N2	WHO CCRRI	This study	Alfred Hospital
EPI_ISL_12851108	A/Victoria/315/2022	3C.2a1b.2a.2	A/H3N2	WHO CCRRI	This study	Monash Medical Centre
EPI_ISL_12851109	A/Victoria/310/2022	3C.2a1b.2a.2	A/H3N2	WHO CCRRI	This study	Monash Medical Centre
EPI_ISL_12851110	A/Victoria/311/2022	3C.2a1b.2a.2	A/H3N2	WHO CCRRI	This study	Monash Medical Centre

EPI_ISL_12851111	A/Victoria/307/2022	3C.2a1b.2a.2	A/H3N2	WHO CCRRI	This study	Monash Medical Centre
EPI_ISL_12851112	A/Victoria/290/2022	3C.2a1b.2a.2	A/H3N2	WHO CCRRI	This study	Monash Medical Centre
EPI_ISL_12851113	A/Victoria/298/2022	3C.2a1b.2a.2	A/H3N2	WHO CCRRI	This study	Monash Medical Centre
EPI_ISL_13168836	A/Victoria/65a/2022	3C.2a1b.2a.2	A/H3N2	WHO CCRRI	This study	Royal Melbourne Hospital
EPI_ISL_13168837	A/Victoria/100/2022	3C.2a1b.2a.2	A/H3N2	WHO CCRRI	This study	Austin Health
EPI_ISL_13168838	A/Victoria/35/2022	3C.2a1b.2a.2	A/H3N2	WHO CCRRI	This study	Alfred Hospital
EPI_ISL_13168839	A/Victoria/36/2022	3C.2a1b.2a.2	A/H3N2	WHO CCRRI	This study	Alfred Hospital
EPI_ISL_13168840	A/Victoria/38/2022	3C.2a1b.2a.2	A/H3N2	WHO CCRRI	This study	Alfred Hospital
EPI_ISL_13168841	A/Victoria/34/2022	3C.2a1b.2a.2	A/H3N2	WHO CCRRI	This study	Austin Health
EPI_ISL_13168842	A/Victoria/75/2022	3C.2a1b.2a.2	A/H3N2	WHO CCRRI	This study	Victorian Infectious Diseases Reference Laboratory
EPI_ISL_13168843	A/Victoria/21/2022	3C.2a1b.2a.2	A/H3N2	WHO CCRRI	This study	Royal Melbourne Hospital
EPI_ISL_13168844	A/Victoria/48/2022	3C.2a1b.2a.2	A/H3N2	WHO CCRRI	This study	ST Vincent Hospital
EPI_ISL_13175618	A/Victoria/984/2022	3C.2a1b.2a.2	A/H3N2	WHO CCRRI	This study	Melbourne Pathology
EPI_ISL_13175622	A/Victoria/993/2022	3C.2a1b.2a.2	A/H3N2	WHO CCRRI	This study	Melbourne Pathology
EPI_ISL_13175639	A/Victoria/811/2022	3C.2a1b.2a.2	A/H3N2	WHO CCRRI	This study	Austin Health
EPI_ISL_13175641	A/Victoria/810/2022	3C.2a1b.2a.2	A/H3N2	WHO CCRRI	This study	Austin Health
EPI_ISL_13175642	A/Victoria/818/2022	3C.2a1b.2a.2	A/H3N2	WHO CCRRI	This study	Austin Health
EPI_ISL_13175651	A/Victoria/1023/2022	3C.2a1b.2a.2	A/H3N2	WHO CCRRI	This study	Melbourne Pathology
EPI_ISL_13175656	A/Victoria/1007/2022	3C.2a1b.2a.2	A/H3N2	WHO CCRRI	This study	Melbourne Pathology
EPI_ISL_13398668	A/Victoria/221/2022	6B.1A.5a.2	A/H1N1	WHO CCRRI	This study	Alfred Hospital
EPI_ISL_13398682	A/Victoria/883/2022	3C.2a1b.2a.2	A/H3N2	WHO CCRRI	This study	Monash Medical Centre
EPI_ISL_13398683	A/Victoria/863/2022	3C.2a1b.2a.2	A/H3N2	WHO CCRRI	This study	Monash Medical Centre
EPI_ISL_13398684	A/Victoria/856/2022	3C.2a1b.2a.2	A/H3N2	WHO CCRRI	This study	Monash Medical Centre
EPI_ISL_13398685	A/Victoria/858/2022	3C.2a1b.2a.2	A/H3N2	WHO CCRRI	This study	Monash Medical Centre
EPI_ISL_13398686	A/Victoria/851/2022	3C.2a1b.2a.2	A/H3N2	WHO CCRRI	This study	Monash Medical Centre
EPI_ISL_13531020	A/Victoria/718/2022	3C.2a1b.2a.2	A/H3N2	WHO CCRRI	This study	Monash Medical Centre
EPI_ISL_13531021	A/Victoria/723/2022	3C.2a1b.2a.2	A/H3N2	WHO CCRRI	This study	Monash Medical Centre
EPI_ISL_13531022	A/Victoria/698/2022	3C.2a1b.2a.2	A/H3N2	WHO CCRRI	This study	Monash Medical Centre
EPI_ISL_13531023	A/Victoria/708/2022	3C.2a1b.2a.2	A/H3N2	WHO CCRRI	This study	Monash Medical Centre
EPI_ISL_13531024	A/Victoria/785/2022	3C.2a1b.2a.2	A/H3N2	WHO CCRRI	This study	Monash Medical Centre
EPI_ISL_13531181	A/Victoria/871/2022	3C.2a1b.2a.2	A/H3N2	WHO CCRRI	This study	Monash Medical Centre
EPI_ISL_13531184	A/Victoria/848/2022	3C.2a1b.2a.2	A/H3N2	WHO CCRRI	This study	Monash Medical Centre
EPI_ISL_13531226	A/Victoria/757/2022	3C.2a1b.2a.2	A/H3N2	WHO CCRRI	This study	Monash Medical Centre
EPI_ISL_13531227	A/Victoria/864/2022	3C.2a1b.2a.2	A/H3N2	WHO CCRRI	This study	Monash Medical Centre
EPI_ISL_13531228	A/Victoria/98/2022	3C.2a1b.2a.2	A/H3N2	WHO CCRRI	This study	Austin Health
EPI_ISL_13531233	A/Victoria/174/2022	3C.2a1b.2a.2	A/H3N2	WHO CCRRI	This study	Melbourne Pathology
EPI_ISL_13531234	A/Victoria/170/2022	3C.2a1b.2a.2	A/H3N2	WHO CCRRI	This study	Melbourne Pathology
EPI_ISL_13531235	A/Victoria/172/2022	3C.2a1b.2a.2	A/H3N2	WHO CCRRI	This study	Melbourne Pathology
EPI_ISL_13531236	A/Victoria/169/2022	3C.2a1b.2a.2	A/H3N2	WHO CCRRI	This study	Melbourne Pathology
EPI_ISL_13655099	A/Victoria/139/2022	3C.2a1b.2a.2	A/H3N2	WHO CCRRI	This study	Monash Medical Centre
EPI_ISL_13655101	A/Victoria/138/2022	3C.2a1b.2a.2	A/H3N2	WHO CCRRI	This study	Monash Medical Centre
EPI_ISL_13655102	A/Victoria/158/2022	3C.2a1b.2a.2	A/H3N2	WHO CCRRI	This study	Monash Medical Centre
EPI_ISL_13655105	A/Victoria/404/2022	3C.2a1b.2a.2	A/H3N2	WHO CCRRI	This study	Alfred Hospital
EPI_ISL_13655108	A/Victoria/519/2022	3C.2a1b.2a.2	A/H3N2	WHO CCRRI	This study	Alfred Hospital
EPI_ISL_13655109	A/Victoria/521/2022	3C.2a1b.2a.2	A/H3N2	WHO CCRRI	This study	Alfred Hospital
EPI_ISL_13655114	A/Victoria/775/2022	3C.2a1b.2a.2	A/H3N2	WHO CCRRI	This study	Monash Medical Centre
EPI_ISL_13655115	A/Victoria/704/2022	3C.2a1b.2a.2	A/H3N2	WHO CCRRI	This study	Monash Medical Centre
EPI_ISL_13983918	A/Victoria/747/2022	6B.1A.5a.2	A/H1N1	WHO CCRRI	This study	Monash Medical Centre
EPI_ISL_13983919	A/Victoria/779/2022	6B.1A.5a.2	A/H1N1	WHO CCRRI	This study	Monash Medical Centre
EPI_ISL_13983978	A/Victoria/532/2022	3C.2a1b.2a.2	A/H3N2	WHO CCRRI	This study	Royal Melbourne Hospital
EPI_ISL_13983982	A/Victoria/936/2022	3C.2a1b.2a.2	A/H3N2	WHO CCRRI	This study	Alfred Hospital
EPI_ISL_13984023	A/Victoria/938/2022	3C.2a1b.2a.2	A/H3N2	WHO CCRRI	This study	Alfred Hospital
EPI_ISL_14115533	A/Victoria/143/2022	3C.2a1b.2a.2	A/H3N2	WHO CCRRI	This study	Royal Melbourne Hospital
EPI_ISL_14161750	A/Victoria/783/2022	3C.2a1b.2a.2	A/H3N2	WHO CCRRI	This study	Monash Medical Centre

EPI_ISL_14161752	A/Victoria/758/2022	3C.2a1b.2a.2	A/H3N2	WHO CCRRI	This study	Monash Medical Centre
EPI_ISL_14161754	A/Victoria/789/2022	3C.2a1b.2a.2	A/H3N2	WHO CCRRI	This study	Monash Medical Centre
EPI_ISL_14161756	A/Victoria/844/2022	3C.2a1b.2a.2	A/H3N2	WHO CCRRI	This study	Monash Medical Centre
EPI_ISL_14161758	A/Victoria/855/2022	3C.2a1b.2a.2	A/H3N2	WHO CCRRI	This study	Monash Medical Centre
EPI_ISL_14161760	A/Victoria/878/2022	3C.2a1b.2a.2	A/H3N2	WHO CCRRI	This study	Monash Medical Centre
EPI_ISL_14161764	A/Victoria/519/2022	3C.2a1b.2a.2	A/H3N2	WHO CCRRI	This study	Alfred Hospital
EPI_ISL_14161766	A/Victoria/521/2022	3C.2a1b.2a.2	A/H3N2	WHO CCRRI	This study	Alfred Hospital
EPI_ISL_14161794	A/Victoria/82/2022	3C.2a1b.2a.2	A/H3N2	WHO CCRRI	This study	Monash Medical Centre
EPI_ISL_14161797	A/Victoria/88/2022	3C.2a1b.2a.2	A/H3N2	WHO CCRRI	This study	Monash Medical Centre
EPI_ISL_14161799	A/Victoria/104/2022	3C.2a1b.2a.2	A/H3N2	WHO CCRRI	This study	Royal Melbourne Hospital
EPI_ISL_14161802	A/Victoria/210/2022	3C.2a1b.2a.2	A/H3N2	WHO CCRRI	This study	Alfred Hospital
EPI_ISL_14161805	A/Victoria/213/2022	3C.2a1b.2a.2	A/H3N2	WHO CCRRI	This study	Alfred Hospital
EPI_ISL_14161808	A/Victoria/212/2022	3C.2a1b.2a.2	A/H3N2	WHO CCRRI	This study	Alfred Hospital
EPI_ISL_14161811	A/Victoria/211/2022	3C.2a1b.2a.2	A/H3N2	WHO CCRRI	This study	Alfred Hospital
EPI_ISL_14161813	A/Victoria/214/2022	3C.2a1b.2a.2	A/H3N2	WHO CCRRI	This study	Alfred Hospital
EPI_ISL_14161816	A/Victoria/215/2022	3C.2a1b.2a.2	A/H3N2	WHO CCRRI	This study	Alfred Hospital
EPI_ISL_14161819	A/Victoria/216/2022	3C.2a1b.2a.2	A/H3N2	WHO CCRRI	This study	Alfred Hospital
EPI_ISL_14161821	A/Victoria/508/2022	3C.2a1b.2a.2	A/H3N2	WHO CCRRI	This study	Alfred Hospital
EPI_ISL_14161833	A/Victoria/760/2022	3C.2a1b.2a.2	A/H3N2	WHO CCRRI	This study	Monash Medical Centre
EPI_ISL_14189363	A/Victoria/772/2022	3C.2a1b.2a.2	A/H3N2	WHO CCRRI	This study	Monash Medical Centre
EPI_ISL_14467656	A/Newcastle/47/2022	3C.2a1b.2a.2	A/H3N2	WHO CCRRI	This study	John Hunter Hospital
EPI_ISL_14819723	A/Victoria/220/2022	3C.2a1b.2a.2	A/H3N2	WHO CCRRI	This study	Alfred Hospital
EPI_ISL_14819724	A/Victoria/231/2022	3C.2a1b.2a.2	A/H3N2	WHO CCRRI	This study	Alfred Hospital
EPI_ISL_15833316	A/Victoria/1414/2022	3C.2a1b.2a.2	A/H3N2	WHO CCRRI	This study	Monash Medical Centre
EPI_ISL_15833317	A/Victoria/1382/2022	3C.2a1b.2a.2	A/H3N2	WHO CCRRI	This study	Monash Medical Centre
EPI_ISL_15833389	A/Victoria/1406/2022	3C.2a1b.2a.2	A/H3N2	WHO CCRRI	This study	Monash Medical Centre
EPI_ISL_15833411	A/Victoria/2609/2022	3C.2a1b.2a.2	A/H3N2	WHO CCRRI	This study	Monash Medical Centre
EPI_ISL_9673931	A/Victoria/7/2021	3C.2a1b.2a.2	A/H3N2	WHO CCRRI	This study	Monash Medical Centre
EPI_ISL_9673932	A/Victoria/9/2021	3C.2a1b.2a.2	A/H3N2	WHO CCRRI	This study	Alfred Hospital
EPI_ISL_9847985	A/Victoria/11/2021	3C.2a1b.2a.2	A/H3N2	WHO CCRRI	This study	Royal Melbourne Hospital
EPI_ISL_9847986	A/Victoria/16/2021	3C.2a1b.2a.2	A/H3N2	WHO CCRRI	This study	Royal Melbourne Hospital
EPI_ISL_9847988	A/Victoria/7/2021	3C.2a1b.2a.2	A/H3N2	WHO CCRRI	This study	Monash Medical Centre

[a]World Health Organisation for Reference and Research on Influenza (WHO CCRRI)

Supplementary Table S4. GISAID Accession numbers for international and reference influenza viruses displayed in the phylogenetic tree (Figure 3)

Isolate_Id	Isolate_Name	Submitting_Lab	Authors	Originating_Lab
EPI_ISL_13044336	A/Tumbes/FPT02314/2022(H3N2)	NAMRU-6[a]	Gilda Troncos, Victoria Espejo, Cristhopher Cruz, Victor Herrera Sunci?n , Yeny Tinoco, Sonia Ampuero, Marita Silva, Paul Graf.	NAMRU-6
EPI_ISL_13044334	A/Tumbes/FPT02308/2022(H3N2)	NAMRU-6	Gilda Troncos, Victoria Espejo, Cristhopher Cruz, Victor Herrera Sunci?n , Yeny Tinoco, Sonia Ampuero, Marita Silva, Paul Graf.	NAMRU-6
EPI_ISL_11171190	A/Tumbes/FPT02244/2021(H3N2)	NAMRU-6	Gilda Troncos, Victoria Espejo, Cristhopher Cruz, Victor Herrera Sunci?n , Yeny Tinoco, Sonia Ampuero, Marita Silva, Paul Graf.	NAMRU-6
EPI_ISL_11171179	A/Bagua/FPG00828/2022(H3N2)	NAMRU-6	Gilda Troncos, Victoria Espejo, Cristhopher Cruz, Jorge Miguel Alexander Olivos Villanueva, Ronald Eduardo Ramos Neyra , Yeny Tinoco, Sonia Ampuero, Marita Silva, Paul Graf.	NAMRU-6
EPI_ISL_11400612	A/Michigan/UOM10043998503/2022	University of Michigan		University of Michigan Clinical Microbiology Laboratory
EPI_ISL_6902173	A/Michigan/UOM10042604678/2021	University of Michigan		University of Michigan Clinical Microbiology Laboratory
EPI_ISL_13148944	A/Human/New York/PV60633/2022	Icahn School of Medicine at Mount Sinai	Gonzalez-Reiche, Ana S.; Alshammary, Hala; Polanco, Jose; Kim, Eunhye; Amoako, Angela; Rooker, Aria; Cognigni, Christian; Floda, Daniel; van de Guchte, Adriana; Khalil, Zain; Srivastava, Komal; Sebra, Robert; Garc?a-Sastre, Adolfo; Patel, Gopi; Schaefer, Sarah; Ramirez, Juan David; Banu, Radhika; Shrestha, Paras; Paniz-Mondolfi, Alberto; Sordillo, Emilia Mia; Simon, Viviana; van Bakel, Harm	Icahn School of Medicine at Mount Sinai
EPI_ISL_8769986	A/Denmark/17/2021	Statens Serum Institute	Trebbien, Ramona; Nissen, Jakob; Bolt Botnen, Amanda	Statens Serum Institute
EPI_ISL_8694331	A/Denmark/39/2021	Statens Serum Institute	Trebbien, Ramona; Nissen, Jakob; Bolt Botnen, Amanda	Statens Serum Institute
EPI_ISL_12323526	A/Denmark/288/2022	Statens Serum Institute	Trebbien, Ramona; Nissen, Jakob; Bolt Botnen, Amanda	Statens Serum Institute
EPI_ISL_13326477	A/Austria/MUW_1510488/2022	Medical University of Vienna	Camp, J.V.; Redlberger-Fritz, M.	Department of Virology, Medical University Vienna
EPI_ISL_11403058	A/Lisboa/23/2022	Instituto Nacional de Saúde Dr. Ricardo Jorge	Guiomar, R.; Melo, A.; Costa, I.; Conde, P, Henriques, C.	Instituto Nacional de Saúde Dr. Ricardo Jorge
EPI_ISL_9775384	A/Luxembourg/LNS6784733/2021	Laboratoire National de Santé	Anke Wienecke-Baldacchino, Sibel Berger, Guillaume Fournier	Laboratoire National de Santé
EPI_ISL_13431097	A/England/220840504/2022	UK Health Security Agency - Colindale	UKHSA, Respiratory Virus Unit	UK Health Security Agency - Colindale
EPI_ISL_9383724	A/England/215121919/2021	UK Health Security Agency - Colindale	UKHSA, Respiratory Virus Unit	UK Health Security Agency - Colindale
EPI_ISL_5935472	A/England/214100500/2021	UK Health Security Agency - Colindale		UK Health Security Agency - Colindale
EPI_ISL_7607226	A/Netherlands/10148/2021	National Institute for Public Health and the Environment (RIVM)	A,Meijer; S, vd Brink; L,Wijsman	PAMM
EPI_ISL_12064265	A/Netherlands/10905/2022	National Institute for Public Health and the Environment (RIVM)	A,Meijer; L,Wijsman; S, vd Brink	Diakonessenhuis Utrecht
EPI_ISL_9627675	A/Israel/S-1/2021	Crick Worldwide Influenza Centre		Central Virology Laboratory Israel (NIC)
EPI_ISL_9533449	A/Israel/R-724/2022	Crick Worldwide Influenza Centre		Central Virology Laboratory Israel (NIC)
EPI_ISL_9842667	A/Norway/521/2022	Crick Worldwide Influenza Centre		WHO National Influenza Centre
EPI_ISL_12942424	A/Ghana/195/2022	Crick Worldwide Influenza Centre		University of Ghana
EPI_ISL_9593247	A/Iran/15/Azar21/2021	Crick Worldwide Influenza Centre		Tehran University of Medical Sciences
EPI_ISL_9593240	A/Iran/83/Ab/8A/2021	Crick Worldwide Influenza Centre		Tehran University of Medical Sciences
EPI_ISL_12219314	A/Serbia/4/2021	Crick Worldwide Influenza Centre		Institute of Immunology and Virology Torlak
EPI_ISL_13017096	A/Morocco/82/2021	Crick Worldwide Influenza Centre		Institut National d'Hygiène
EPI_ISL_13339904	A/Ireland/17354/2022	Crick Worldwide Influenza Centre		UCD National Virus Reference Laboratory
EPI_ISL_9842672	A/Ireland/2795/2022	Crick Worldwide Influenza Centre		UCD National Virus Reference Laboratory

EPI_ISL_8808670	A/Ireland/92285/2021	Crick Worldwide Influenza Centre		UCD National Virus Reference Laboratory
EPI_ISL_8808663	A/Ireland/82/2021	Crick Worldwide Influenza Centre		UCD National Virus Reference Laboratory
EPI_ISL_13203500	A/Iceland/28760/2022	Crick Worldwide Influenza Centre		Landspitali - University Hospital
EPI_ISL_13203460	A/Iceland/33333/2022	Crick Worldwide Influenza Centre		Landspitali - University Hospital
EPI_ISL_9627689	A/Romania/495025/2021	Crick Worldwide Influenza Centre		Cantacuzino Institute
EPI_ISL_9593243	A/Hungary/8/2021	Crick Worldwide Influenza Centre		Central Veterinary Institute Budapest
EPI_ISL_9593233	A/Hungary/2/2022	Crick Worldwide Influenza Centre		Central Veterinary Institute Budapest
EPI_ISL_12219613	A/Bosnia and Herzegovina/103/2021	Crick Worldwide Influenza Centre		University of Sarajevo
EPI_ISL_12219468	A/Cote d'Ivoire/4032/2021	Crick Worldwide Influenza Centre		Pasteur Institut of Côte d'Ivoire
EPI_ISL_12219451	A/Cote d'Ivoire/4120/2021	Crick Worldwide Influenza Centre		Pasteur Institut of Côte d'Ivoire
EPI_ISL_12219432	A/Cote d'Ivoire/8/2022	Crick Worldwide Influenza Centre		Pasteur Institut of Côte d'Ivoire
EPI_ISL_12219308	A/Kosova/954/2022	Crick Worldwide Influenza Centre		National Institute of Public Health of Kosova
EPI_ISL_12240159	A/Netherlands/10024/2021	Crick Worldwide Influenza Centre		National Institute for Public Health and the Environment (RIVM)
EPI_ISL_13203484	A/Switzerland/83625/2022	Crick Worldwide Influenza Centre		Swiss National Reference Centre for Influenza
EPI_ISL_9401999	A/Switzerland/69954/2021	Crick Worldwide Influenza Centre		Swiss National Reference Centre for Influenza
EPI_ISL_12839635	A/Sachsen/7/2022	Robert Koch Institute Nationales Referenzzentrum für Influenza		Robert Koch-Institute Nationales Referenzzentrum für Influenza
EPI_ISL_11217722	A/Hessen/4/2022	Robert Koch Institute Nationales Referenzzentrum für Influenza		Robert Koch-Institute Nationales Referenzzentrum für Influenza
EPI_ISL_13812784	A/United Kingdom/13176/2022	U.S. Air Force School of Aerospace Medicine	Gruner, W.E., Muehleman, D.M., Fries, A.C., Garrett, C.M., Hanson, J.F., DeMarcus, L.S., Sjoberg, P.A., Wasik, P.M., Robbins, A.S., Macias, E.A., Shaw, K.B., Selent, M.U.	U.S. Air Force School of Aerospace Medicine
EPI_ISL_12422440	A/Texas/12644/2021	U.S. Air Force School of Aerospace Medicine	Gruner, W.E., Muehleman, D.M., Fries, A.C., Garrett, C.M., Hanson, J.F., DeMarcus, L.S., Sjoberg, P.A., Wasik, P.M., Robbins, A.S., Macias, E.A., Shaw, K.B., Selent, M.U.	U.S. Air Force School of Aerospace Medicine
EPI_ISL_7404317	A/Maryland/12435/2021	U.S. Air Force School of Aerospace Medicine	Gruner, W.E., Muehleman, D.M., Fries, A.C., Garrett, C.M., Hanson, J.F., DeMarcus, L.S., Sjoberg, P.A., Wasik, P.M., Robbins, A.S., Macias, E.A., Shaw, K.B., Selent, M.U.	U.S. Air Force School of Aerospace Medicine
EPI_ISL_9994834	A/Catalonia/NSVH101718786/2022	Hospital Universitari Vall d'Hebron	Anton, A.; Martin, MC.; Andres, C.; Codina, MG.; Pumarola, T.	Hospital Universitari Vall d'Hebron
EPI_ISL_12609685	A/Romania/509797_BB/2022	Cantacuzino Institute	Luiza,Ustea; Oana,Vitencu; Catalina,Pascu; Sorin,Dinu; Mihaela,Lazar	Cantacuzino Institute
EPI_ISL_12607361	A/Romania/510965_GR/2022	Cantacuzino Institute	Luiza,Ustea; Oana,Vitencu; Catalina,Pascu; Sorin,Dinu; Mihaela,Lazar	Cantacuzino Institute
EPI_ISL_12646189	A/Andalucia/1528/2022	Instituto de Salud Carlos III	Pozo,F; Iglesias-Caballero, M.; Gonz?lez-Esguevillas,M.; Molinero,M.; Camarero,S.; Mart?n,V.; Reyes,N.; Sandon?s,V.; V?zquez-Mor?n,S.; Casas,I.	Servicio de Microbiología Hospital Virgen de las Nieves
EPI_ISL_10337732	A/Netherlands/00090/2021	Erasmus Medical Center		Erasmus Medical Center
EPI_ISL_9760141	A/Netherlands/00048/2022	Erasmus Medical Center		Erasmus Medical Center
EPI_ISL_14033333	A/Guizhou-Anlong/319/2022	WHO Chinese National Influenza Center	Zeng xiaoxu, Li Xiyan, Huang Weijuan, Dayan Wang	WHO Chinese National Influenza Center
EPI_ISL_14033290	A/Guizhou-Liuzhite/326/2022	WHO Chinese National Influenza Center	Zeng xiaoxu, Li Xiyan, Huang Weijuan, Dayan Wang	WHO Chinese National Influenza Center
EPI_ISL_14033215	A/Guangxi-Fangcheng/11331/2021	WHO Chinese National Influenza Center	Zeng xiaoxu, Li Xiyan, Huang Weijuan, Dayan Wang	WHO Chinese National Influenza Center
EPI_ISL_13440481	A/YOKOHAMA/3/2021	National Institute of Infectious Diseases	Takashita,Emi;Fujisaki,Seiichiro;Watanabe,Shinji;Hasegawa,Hideki	Yokohama City Institute of Public Health.
EPI_ISL_13284206	A/Colombia/2496/2022	CDC USA[b]		Instituto Nacional de Salud de Columbia
EPI_ISL_13580811	A/El Salvador/9212/2022	CDC USA		Contiguo a Hospital Rosales
EPI_ISL_12710455	A/Madagascar/3048/2021	CDC USA		Institut Pasteur de Madagascar
EPI_ISL_13892666	A/Serbia/2189/2022	CDC USA		Institute of Immunology and Virology Torlak

EPI_ISL_13892659	A/Serbia/2262/2022	CDC USA		Institute of Immunology and Virology Torlak
EPI_ISL_8808407	A/Abu Dhabi/3550476/2021	CDC USA		Shaikh Khalifa Medical City
EPI_ISL_13284263	A/Argentina/2214/2022	CDC USA		Instituto Nacional de Enfermedades Infecciosas
EPI_ISL_10828452	A/India/Pun-NIV60/2021	CDC USA		National Institute of Virology
EPI_ISL_10826393	A/Kenya/101/2022	CDC USA		CDC-Kenya
EPI_ISL_13284264	A/Nicaragua/2624/2022	CDC USA		Laboratorio de Virologia, Direccion de Microbiologia
EPI_ISL_9346127	A/Pakistan/ICT12/2021	CDC USA		National Institute of Health
EPI_ISL_9346122	A/Pakistan/KE3/2021	CDC USA		National Institute of Health
EPI_ISL_13284202	A/Uruguay/127/2022	CDC USA		Departamento de Laboratorio de Salud Pública (DLSP)
EPI_ISL_14036331	A/Guyane/365/2021	CDC USA		National Influenza Center French Guiana and French Indies
EPI_ISL_13343701	A/Puerto Rico/02/2022	CDC USA		Puerto Rico Department of Health
EPI_ISL_13284051	A/Puerto Rico/06/2022	CDC USA		Puerto Rico Department of Health
EPI_ISL_10493003	A/Puerto Rico/04/2021	CDC USA		Puerto Rico Department of Health
EPI_ISL_10827595	A/Minnesota/06/2022	CDC USA		Minnesota Department of Health
EPI_ISL_11969459	A/Congo/026/2022	CDC USA		INRB Service de Virologie
EPI_ISL_9388974	A/Ethiopia/0603/2021	CDC USA		Ethiopian Public Health Institute, National Influenza Laboratory
EPI_ISL_9389004	A/Bangladesh/4037/2021	CDC USA		icddr,b International Centre for Diarrhoeal Disease Research, Bangladesh
EPI_ISL_13892749	A/Dominican Republic/195/2022	CDC USA		Laboratorio Nacional de Salud Publica Dr. Defillo
EPI_ISL_13284698	A/Niger/10289/2022	CDC USA		Centre de Recherche Medicale et Sanitaire (CERMES)
EPI_ISL_11956882	A/Nigeria/6593/2021	CDC USA		NCDC Public Health Reference Laboratory
EPI_ISL_13655578	A/Singapore/KK0015/2022	Ministry of Health, Singapore	Chen,B.;Zhou, Z.; Yeo,B.; Cui,L; Lin,R.T.P.	Ministry of Health, Singapore
EPI_ISL_8640747	A/Singapore/INFTT0006/2021	Ministry of Health, Singapore	Chen,B.;Zhou,Z.; Ching,K.C.; Cui,L; Lin,R.T.P.	Ministry of Health, Singapore
EPI_ISL_9067303	A/Sweden/15/2021	Public Health Agency of Sweden		Public Health Agency of Sweden
EPI_ISL_12809147	A/Lorraine/29637/2022	Institut Pasteur		Institut Pasteur
EPI_ISL_11323853	A/PAPEETE/51874/2021	Institut Pasteur		Institut Pasteur

[a]Naval Medical Research Unit 6 (NAMRU-6)

[b]Centers for Disease Control and Prevention

Supplementary Table S5. Comparison of demographics between interviewed and non-interviewed influenza cases, Victoria, Australia, 1 November 2021 – 6 April 2022

	Interviewed (n = 124)		Not interviewed (n = 76)		p-value[a]
	n	%	n	%	
Sex					0.51
- Female	66	53.2%	36	47.4%	
- Male	58	46.8%	40	52.6%	
Age group (years)					0.86
- <5	6	4.8%	3	3.9%	
- 5-14	12	9.7%	5	6.6%	
- 15-24	66	53.2%	45	59.2%	
- 25-34	22	17.7%	12	15.8%	
- 35-44	1	0.8%	3	3.9%	
- 45-54	7	5.6%	3	3.9%	
- 55-64	3	2.4%	2	2.6%	
- 65-74	3	2.4%	1	1.3%	
- ≥75	4	3.2%	2	2.6%	
- Total	124	100.0%	76	100.0%	

[a]p-values calculated with chi-squared tests, excluding unknown values. Based on inclusion of unknown values, p=<0.001 for both age and sex.

Supplementary Figure S1. Estimates of the proportion of total influenza cases exposed to international travel, based on the subset of interviewed cases, Victoria, Australia, 1 November 2021 – 6 April 2022.

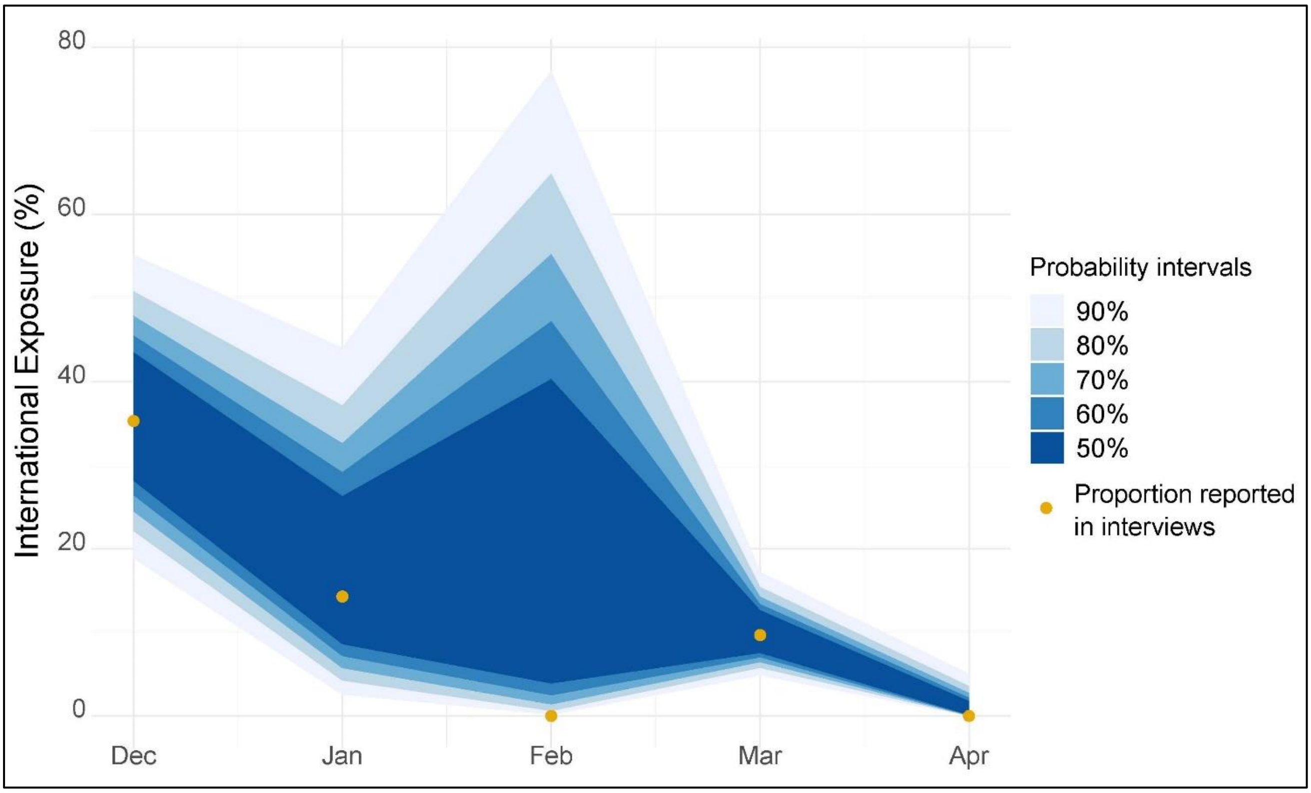

This plot shows calculated credible intervals across a range of probabilities (50-90%) for international travel exposure among all cases based on the assumption that the proportion of interviewed cases who reported an international exposure is a binomial process. Uncertainty of these estimates was higher in months with lower numbers of interviewed cases, most notably February. Data source: Department of Health and interviews conducted in this study.

Supplementary Table S6. Health service attendance and pathways to accessing influenza testing data from case interviews for first 200 influenza notifications (n=124), Victoria, Australia, 1 November 2021 – 6 April 2022

	First accessed		Tested		Accessed	
	n	%	n	%	n	%
GP	55	45.1	43	35.2	66	54.1
ED	30	24.6	52	42.6	56	45.9
COVID testing centre	23	20.5	16	13.1	26	21.3
Ambulance	2	1.6	0	0.0	9	7.4
Nurse-on-call[a]	5	4.1	0	0.0	7	5.7
Workplace	2	1.6	3	2.5	3	2.5
Specialist outpatient	1	0.8	1	0.8	1	0.8
Inpatient	1	0.8	4	3.3	3	2.5

[a]Nurse-led telehealth advice service.

Supplementary Figure S2. Cluster diagram of influenza cases.

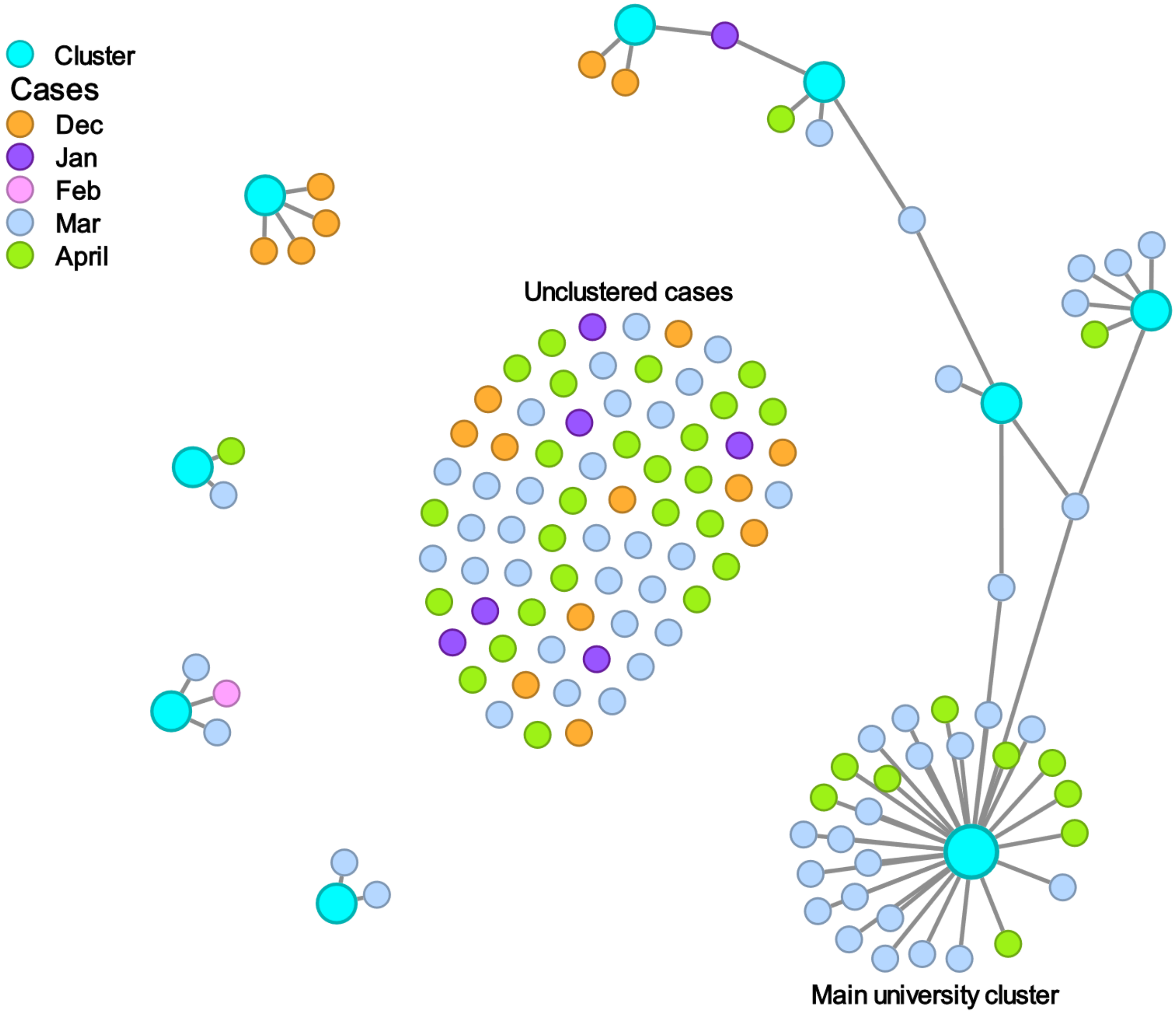

This figure is a representation of patterns of spread within the population. Clusters defined by common influenza exposures between interviewed cases. Only interviewed cases are depicted. Detailed contact tracing was not performed and in most instances it was not possible to identify direct person-to-person transmission between interviewed cases, based on these limitations. Data source: interviews conducted in this study.
